# Unveiling the human fetal-maternal interface during the first trimester: biophysical knowledge and gaps

**DOI:** 10.3389/fcell.2024.1411582

**Published:** 2024-07-31

**Authors:** Alice Masserdotti, Michael Gasik, Regina Grillari-Voglauer, Johannes Grillari, Anna Cargnoni, Paola Chiodelli, Andrea Papait, Marta Magatti, Jacopo Romoli, Sara Ficai, Lorena Di Pietro, Wanda Lattanzi, Antonietta Rosa Silini, Ornella Parolini

**Affiliations:** ^1^ Department of Life Science and Public Health, Università Cattolica del Sacro Cuore, Rome, Italy; ^2^ Seqvera Ltd., Helsinki, Finland; ^3^ Evercyte GmbH, Vienna, Austria; ^4^ Ludwig Boltzmann Institute for Traumatology, The Research Center in Cooperation with AUVA, Vienna, Austria; ^5^ Institute of Molecular Biotechnology, BOKU University, Vienna, Austria; ^6^ Austrian Cluster for Tissue Regeneration, Austria; ^7^ Centro di Ricerca E. Menni, Fondazione Poliambulanza Istituto Ospedaliero, Brescia, Italy; ^8^ Fondazione Policlinico Universitario “Agostino Gemelli” IRCCS, Rome, Italy

**Keywords:** placenta, fetal-maternal interactions, first trimester, fetal membranes, metabolite exchanges

## Abstract

The intricate interplay between the developing placenta and fetal-maternal interactions is critical for pregnancy outcomes. Despite advancements, gaps persist in understanding biomechanics, transport processes, and blood circulation parameters, all of which are crucial for safe pregnancies. Moreover, the complexity of fetal-maternal interactions led to conflicting data and methodological variations. This review presents a comprehensive overview of current knowledge on fetal-maternal interface structures, with a particular focus on the first trimester. More in detail, the embryological development, structural characteristics, and physiological functions of placental chorionic plate and villi, fetal membranes and umbilical cord are discussed. Furthermore, a description of the main structures and features of maternal and fetal fluid dynamic exchanges is provided. However, ethical constraints and technological limitations pose still challenges to studying early placental development directly, which calls for sophisticated *in vitro*, microfluidic organotypic models for advancing our understanding. For this, knowledge about key *in vivo* parameters are necessary for their design. In this scenario, the integration of data from later gestational stages and mathematical/computational simulations have proven to be useful tools. Notwithstanding, further research into cellular and molecular mechanisms at the fetal-maternal interface is essential for enhancing prenatal care and improving maternal and fetal health outcomes.

## 1 Introduction

A thorough understanding of structure, functions, and mechanical features of developing human placenta provides the basis for examining fetal-maternal interactions *in vitro* and *in vivo* that can shed light on potential health outcomes for both the mother and the fetus, and where especially *in vitro* organotypic cultures also allow for testing interventional strategies. Such research is also vital for the identification and mitigation of any risks or complications that may arise during pregnancy. In addition, a thorough understanding of these interactions enables the development of strategies for preventing and intervening in cases where complications or adverse effects may occur.

Central to this is understanding how the fetal-maternal interactions evolve alongside the dynamic changes in placental anatomy and physiology throughout pregnancy’s stages. Research in this domain stands as a cornerstone of medical progress, paving the way for innovations in prenatal care, diagnostics, and treatments that promise to elevate maternal and fetal health outcomes. Yet, these improvements depend on a wide understanding of the complexities of the placenta.

This review is focused on gathering published data essential for understanding fetal-maternal interactions with a particular focus on the first trimester of pregnancy. The intent is to shed light on various aspects of placental biology, exploring the intricate processes involved in its formation and growth, the structural characteristics that define its functionality, and the mechanical properties that contribute to its role in supporting fetal development. Thus, the review will report for each relevant placenta tissue, the development, the structure, the functions and all features and dimensions found in literature, to the best of our knowledge, useful to describe fetal-maternal interface at that tissue level. With the progress of increasingly advanced technologies, strides have been made in understanding some aspects of the placenta development during this gestational phase. For instance, the imaging techniques improvement enable detailed 3D visualization of several placental portions, facilitating precise mathematical modeling. Mathematical modelling approaches complement experimental findings by allowing hypothesis testing, especially in scenarios where experimental methods are challenging (Tan et al., 2014; Allerkamp et al., 2021). However, gaps persist in our understanding of the first trimester placenta due to ethical constraints on obtaining placental tissues during early stages and limitations posed by fetal size. In such instances, leveraging data from other developmental stages through mathematical/computational simulations offers a promising avenue to bridge these knowledge gaps.

## 2 Chorionic plate and villi

### 2.1 Development and structure of chorionic plate and villi

The initiation of human placental development occurs within the first days following conception, through coordinated processes involving distinct yet complementary fetal and maternal tissues. Fetal structures, such as chorionic plate and villi, fetal membrane and umbilical cord, originate from the blastocyst, while the maternal component derives from the endometrium.

**FIGURE 1:**
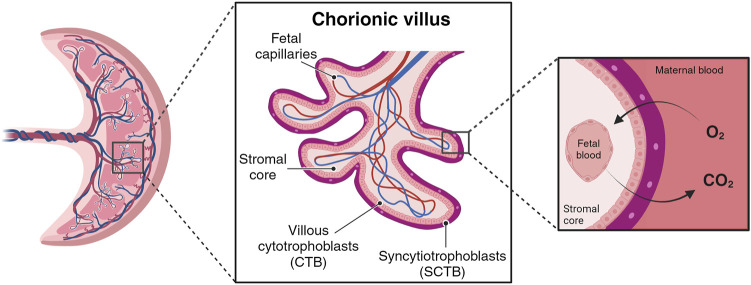
Simplified chorionic villous. Maternal blood flow into the placental intervillous space begins early in the first trimester. The primary barrier for species transport resides in the villous wall, comprising layers such syncytiotrophoblast (SCTB), cytotrophoblast (CTB), and vessels endothelial layers. (Created with BioRender.com).

During the initial week post-implantation, the trophoblast part of the blastocyst undergoes differentiation, giving rise to an outer layer called the syncytiotrophoblast (SCTB) and an inner layer called the cytotrophoblast (CTB). SCTB cells secrete human chorionic gonadotrophic hormone, essential for the maintenance of the corpus luteum during early pregnancy, and form floating finger-like projections from chorionic plate called chorionic villi (Jeschke et al., 2005; Silini et al., 2020). The SCTB cells are the outer layer of villous structure, succeeded by a complete layer of villous CTB cells (Figure 1). The CTB cells have the ability to proliferate and invade the uterine wall where the villous surface comes into contact with the maternal stromal core, forming anchoring villi. These specialized structures secure the expanding placenta to the uterine wall and serve as peripheral trophoblastic adaptations that facilitate the invasion of maternal uterine stroma and blood vessels (Aplin et al., 1998; Moser et al., 2018).

By the conclusion of the third week, mesodermal cells within the villi differentiate into endothelial and blood cells, giving rise to small blood vessels that contribute to the capillary system of the villi (Kojima et al., 2022). Histologically and morphologically, villi progress towards tertiary villi, exhibiting homogeneity and rich mesenchyme with minimal capillaries. These villi, termed mesenchymal villi, possess a diameter of 100–250 μm and represent the most primitive villi with nascent vessels inside (Castellucci et al., 1990). As the villous trees expand, mesenchymal villi undergo longitudinal growth, followed by increased density and width through trophoblast, extra-embryonic mesoblast, and endothelial cell proliferation (Gundacker and Ellinger, 2020). Thus, around week eight, mesenchymal villi transform into immature intermediate villi (100–400 μm in diameter) (Kaufmann et al., 1985; Jauniaux et al., 1991; Jones and Jauniaux, 1995; Huppertz, 2008; Knöfler et al., 2019). Although these villi contain small vessels, they are not optimally suited for nutrient and gas exchange due to the considerable distance between the circulatory units (Leiser et al., 1985; Lisman et al., 2007; Benirschke et al., 2012). The optimal nutrient and gas exchange capabilities only emerge after mid-gestation, coinciding with the appearance of terminal villi around weeks 25–27 of pregnancy, with the development of the terminal villi, characterized by abundant, large diameter vessels that ensure enhanced perfusion (Leiser et al., 1985; Huppertz, 2008).

From a spatial arrangement perspective, trophoblastic villi surround the embryo, creating a fuzzy sphere appearance, until the end of the first trimester. Around week 12, the majority of these placental villi regress, persisting only at the basal plate (Silini et al., 2020). Subsequently, the chorion transforms into a villus-rich chorion (chorion frondosum), a substantial component of the placenta, with larger villi extending into the intervillous space. This transformation involves longitudinal growth, branching, and sprouting, ultimately culminating in the formation of tree-like structures referred to as villous trees. In locations where villi degenerate, the chorion becomes smooth (chorion laeve), consisting of extra-embryonic mesenchyme and CTB, precluding exchange between maternal and fetal circulatory systems (Jones and Jauniaux, 1995; Silini et al., 2020).

The maternal component of placenta is the decidua, formed by the growth and proliferation of functional layer cells in the endometrium after implantation. Based on its relative location to the fetus, the decidua is divided into three regions. The decidua basalis originates from the structural and functional transformation of the endometrium, residing at the site of embryo implantation and interacting with the trophoblasts. The decidua capsularis refers to the part of the decidua that grows over the conceptus on the luminal side. The remaining section of the endometrium is the decidua parietalis, covering the opposite wall of the uterus and merging with the decidua capsularis by the fourth month of gestation (Abomaray et al., 2016; Abumaree et al., 2016).

**FIGURE 2:**
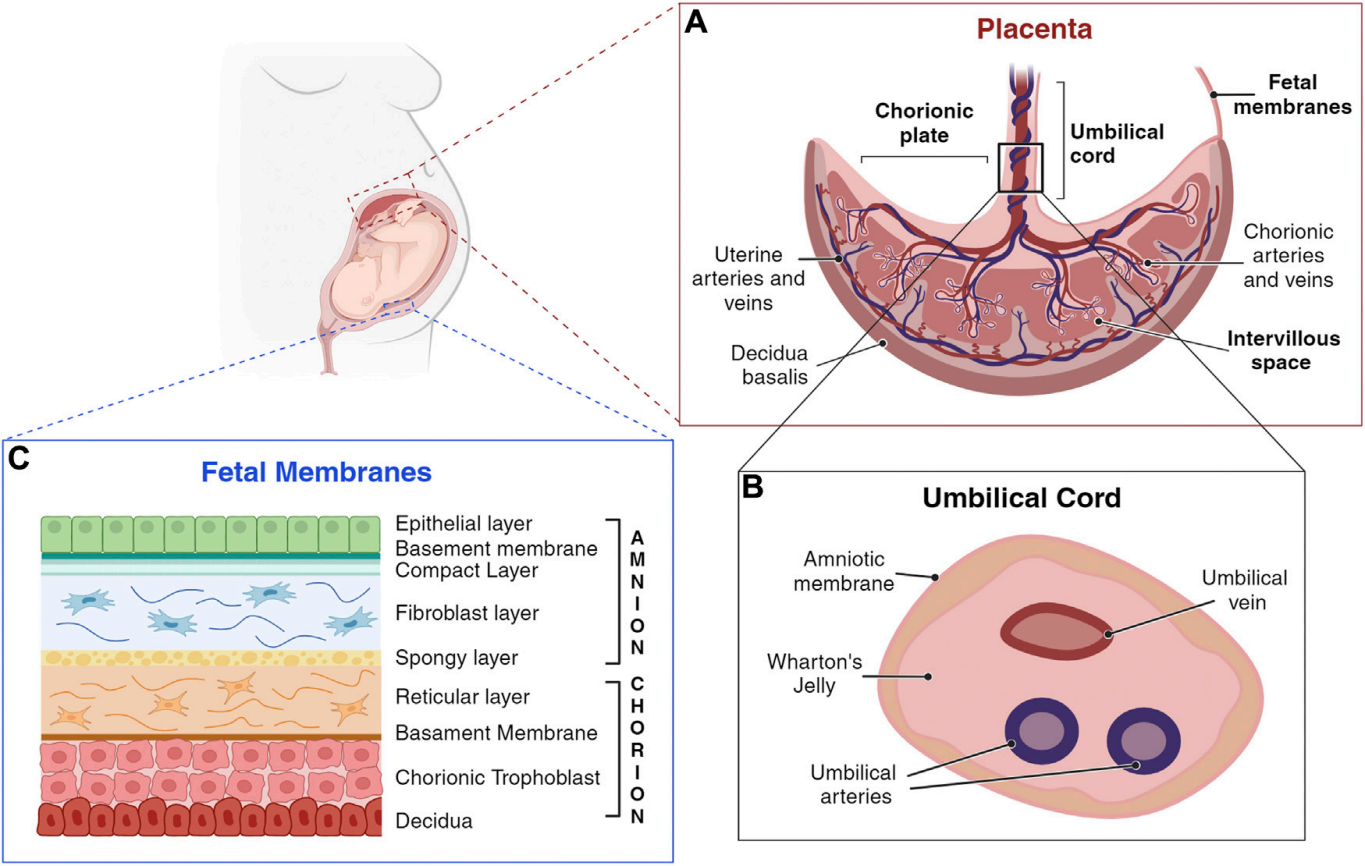
Schematic representation of a human placenta, umbilical cord, and fetal membranes. **(A)** Umbilical cord anchors into the chorionic plate, the fetal component of the placenta, from which placental villi grow into the intervillous space. The maternal component of the placenta consists of decidua, which interacts with trophoblasts at the implantation site. **(B)** The umbilical cord is a flexible stalk connecting the developing embryo or fetus to the placenta. It contains one vein and two arteries, surrounded by Wharton’s jelly and encased in an amniotic sheath. **(C)** Amnion and chorion are considered placental annexes, each one characterized by different layers composition (Silini et al., 2020). (Created with BioRender.com).

During the first 3 months of pregnancy several placenta components undergo dramatic structural and functional changes, fulfilling the needs of the developing fetus (Huppertz et al., 2014). However, within the first trimester of pregnancy, the organization and structure of the placenta is established (Figure 2, panel A) (Huppertz et al., 2014).

### 2.2 Functional aspects of chorionic plate and villi

Several distinct structures are discernible in the first trimester placenta, essential for ensuring an adequate supply of nutrients to the developing embryo. First trimester villi, including mesenchymal villi, immature intermediate villi, and anchoring villi collectively contribute to the expansion of the villous surface, enabling efficient transport for exchange. The resulting first trimester villous surface area is approximately 1–1.5 m^2^, resulting in a specific surface area of 600–800 cm^2^/mL of intervillous space volume (Aherne and Dunnill, 1966; Boyd and Hamilton, 1967; McNanley, 2008). This was reported to correspond to about 92% of apparent porosity (i.e. the volume of intervillous space) at the end of the first trimester, despite the presence of numerous inconsistencies between studies (Lecarpentier et al., 2016; James et al., 2018b; Plitman Mayo, 2018). It is noteworthy that, in the absence of fully differentiated terminal villi, the immature intermediate villi are the main sites of exchange during the first and second trimesters. However, during much of the first trimester, the intervillous spaces are not yet adequately filled with maternal blood and nutritional support of the embryo is primarily assured by substances from maternal plasma, and by products secreted by the uterine glands. This has been termed histotrophic nutrition by glands rather than vessels (Burton et al., 2002; Huppertz, 2019; Huppertz, 2020). Indeed, the placenta functions also as a dynamic endocrine organ, releasing several factors into the maternal bloodstream to facilitate fetal growth and development. The SCTB cells secrete steroids, glycoproteins, peptide hormones, cytokines, extracellular vesicles including miRNA among other cargo (Redman and Sargent, 2008), and neuroactive factors. All these secreted factors play a crucial role in various processes, such as averting immune rejection of the fetus, adjusting maternal metabolic, renal, respiratory, neuronal, and cardiovascular functions to meet the demands of nutrient and oxygen supply to the pregnant uterus, and preparing the mother for lactational support of her child (Gude et al., 2004).

The multinucleated syncytial layer of villi acts as a physical and metabolic semipermeable barrier/filter between the fetal and maternal blood circulations (Stulc, 1989). Passive diffusion is the main mechanism of transfer from maternal blood to fetal blood across this single layer of epithelial cells (Hay, 1994), with an estimated villous diffusion in the third trimester of 0.9 × 10^−9^ m^2^/s (assuming purely diffusive oxygen transport in the villous wall) and 1.67 × 10^−9^ m^2^/s in the fluid (Burton et al., 2009; Chernyavsky et al., 2010; Perazzolo et al., 2017). As described below, other transport systems contribute to nutrient supply to the fetus (Walker et al., 2017), for example active systems such as the amino acid transport (Jansson, 2001). It is expected, however, that the transport conditions are largely affected by local hemodynamic conditions, pressure differences, flow rates and possible turbulence (Chernyavsky et al., 2011). Dramatic changes in ion fluxes during different stages of placenta development were described in *in vitro* (Jones and Fox, 1991) and *in vivo* (Edwards et al., 1993) studies. For instance, cytotrophoblast cell differentiation stages affect membrane potential (E_m_) and the expression of ion transport proteins. The first trimester E_m_ is significantly hyperpolarized compared with that at term, showing −28 mV and −21 mV, respectively. Moreover, the magnitude of the depolarization is greater in first trimester than at term, indicating that the ion conductance of the microvillous membrane (MVM) potential changes with gestation. The major contributor to the change in the magnitude of E_m_ is the alteration in the relative K^+^:Cl^−^conductance of the MVM, finally affecting the formation of the placenta at different stages (Birdsey et al., 1997).

### 2.3 Biophysical features and dimensions of interface

To optimally carry out its functions, the placenta exhibits specific biomechanical characteristics. During early pregnancy, when nutrition is primarily supported by glands, the portion of placenta that acts as a barrier at the fetal-maternal interface is notably thick, ranging from 20 to 30 μm and exhibiting a bilayered structure (Huppertz, 2008; Aplin et al., 2009). As pregnancy progresses, the placental interface undergoes a transformation, becoming thinner, typically ranging from 2 to 4 μm (Feneley and Burton, 1991; Włoch et al., 2009), and predominantly monolayered. This thinning is primarily attributed to the partial disappearance of the CTB layer (Huppertz, 2008; Aplin et al., 2009). The placental total surface area tends to gradually increase with gestational age (to approximately 12 m^2^), at the same pace as fetal growth and development (El Behery et al., 2011).

At the end of the first trimester, the entire human placenta typically has a thickness of 14 ± 2 mm (Karthikeyan et al., 2012; Tiwari and Chandnani, 2013; Vachon-Marceau et al., 2017; Ahmed et al., 2023) and a total volume ranging about 90–110 mL (de Paula et al., 2008). This consolidated structure results from the contributions of 15–30 cotyledons, which would be represented by sectors of about 10–15 × 20 mm size, if assumed to be uniformly distributed across the whole volume (de Paula et al., 2008; Saghian et al., 2017b).

One crucial adaptation is the placenta’s ability to remodel the entire uterine vasculature to accommodate a 15-fold increase in uterine blood flow towards its surface during pregnancy, ensuring adequate fetal-maternal exchange (Ahmed et al., 2023). This process commences with anchoring villi, where CTB cells proliferate, giving rise to extravillous trophoblast (EVT) cells that invade the maternal decidua. EVT cells acquire an endovascular trophoblast phenotype, colonizing and remodeling the spiral arteries (SAs) by destroying the muscle layer and replacing the endothelial lining with a pseudo-endothelium of fetal origin, thus originating wide non-muscular vessels independent of maternal vasoconstriction (Pijnenborg et al., 1981). This vascular remodeling leads to the so called “plugs” in SAs lumen. Haemodynamically this process is crucial in decreasing the speed and pressure with which the blood enters the intervillous space (IVS). This allows maternal blood to evenly perfuse the placental villi and remain in the IVS for an optimal time for oxygen and nutrient transfer between maternal and fetal circulations (Burton et al., 2009; Saghian et al., 2017a). The diameter of maternal SAs expands from 0.2 to 0.25 mm (6–8 weeks), 0.3–0.4 mm (12–14 weeks) to 1–2 mm at the intervillous space level (Harris and Ramsey, 1966; Allerkamp et al., 2021), however, there are different data regarding the flow rate and pressure variations (Burton et al., 2009; Collins et al., 2012; Lecarpentier et al., 2016; James et al., 2018b; Martin et al., 2020).

It is interesting to underline that vascular remodeling precedes the trophoblast invasion of the SAs and is likely initiated by the resident uterine NK cells. These cells, together with the decidual stromal fibroblasts (derived from the differentiation of the endometrial stromal fibroblast cells), promote the functional and morphological changes leading to the formation of decidua basalis (process called also endometrial decidualization), required for blastocyst implantation. The thickness of the decidual layer progressively increases starting from 4 weeks of gestation, reaching its peak at 6–7 weeks, with a measurement of 10 mm. Afterwards, it progressively thins and from 10 weeks of gestation it is no longer measurable (Wong et al., 2009). The stiffness of the decidua basalis can have a role in regulating the differentiation of the decidual stromal fibroblasts and the invasion of the EVT into the decidua. Recently, Abbas and colleagues (Abbas et al., 2019) evaluated the stiffness of the decidua basalis during the first trimester. They found that the apparent elastic modulus of the nonpregnant endometrium at the secretory phase of the menstrual cycle was 250 Pa, not significantly different from that observed for decidua parietalis (171 Pa). In contrast, at 12 weeks of gestation they observed a higher stiffness in the decidua basalis equal to 1250 Pa, a parameter as tissue stiffness not only is important in biomechanical perspectives, but also in terms of cell behavior and differentiation via mechanosensing (Wong et al., 2018). It has to be noted that the stiffness or moduli estimated values depend on the preparation of the sample (if used *ex-vivo*) or signal transforming method (if used from an ultrasound device), data conditioning, the mechanical model used (elastic, hyperelastic, viscoelastic, etc.), fitting parameters and also the exact method of conversion of deformation filed and forces to stress and strain values (Bauer et al., 2009; Chua and Oyen, 2009; Hu et al., 2009), which in the best case should be made model-free (Gasik, 2021).

The onset of maternal flow into intervillous space (IVS) occurs around 7 weeks of gestation, concurrently with the formation of channels within trophoblastic plugs (Roberts et al., 2017; Allerkamp et al., 2021). Moreover, an increase in oxygen tension, from <20 mmHg to >50 mmHg, in the placenta occurred around week 10 (Burton et al., 2002; Onogi et al., 2011). Authors of this work have estimated, based on previous research (Rooth et al., 1961; Kiserud and Acharya, 2004), that the solubility of oxygen (O_2_) in the blood is about 1.36 μM/mmHg and for carbon dioxide (CO_2_) is about 30.1 μM/mmHg. In blood, the solubility of O_2_ and CO_2_ competes with each other. As a result, there exists a negative correlation between O_2_ tension and CO_2_: in fetal arterial blood O_2_ pressure (pO_2_) = 47.05–0.646·CO_2_ pressure (oxygen saturation 50%–60%) and in fetal venous blood pO_2_ = 49.25–0.5642·CO_2_ pressure (oxygen saturation 80%), according to the averaged data (Rooth et al., 1961). The maternal blood residence time in the IVS could be estimated of 25–30 s (for 80 bpm heart rate, this means time needed for about 30–36 pulsations), allowing sufficient time for O_2_ exchange (Kingdom et al., 2020).

## 3 Fetal membranes

### 3.1 Development and structure of fetal membranes

The fetal membranes include the amniotic (amnion) and the chorionic (chorion) membranes (Parolini, 2016), that form a highly specialized and protective interface between the mother and the fetus (Gude et al., 2004; Parolini et al., 2008; Benirschke et al., 2012; Magatti et al., 2019). They enclose the amniotic fluid (AF), a watery liquid filling the amniotic cavity, that provides essential fluids, nutrients, and mechanical protection to the fetus. Fetal membranes development begins promptly after conception and occurs independently of one another (Silini et al., 2020). The amnion, the innermost membrane that encloses the embryo, derives from the inner cell mass, after cells migrate to cover the amniotic cavity (Moore and Persaud, 1998), and is comprised of five layers (Figure 2, Panel C). The closest layer to the fetus is composed of cuboidal and columnar epithelial cells forming an uninterrupted monolayer constituting the amniotic epithelium (1). The ultrastructure of amniotic epithelial cells suggests active secretory and transport functions (Gupta et al., 2015), also supported by the presence of many microvilli at their apical surface (Pollard et al., 1976; Basile et al., 2023), and by the expression of different aquaporin channels (Prat et al., 2012; Ducza et al., 2017). These cells are firmly attached to the basement membrane (2), rich of collagen type IV subchain (Kanayama et al., 1985). The basement membrane, in turn, is affixed to a condensed acellular layer of amniotic mesoderm, the compact layer (3), comprising collagen types I, II, and V (Calvin and Oyen, 2007; Jabareen et al., 2009; Gupta et al., 2015). Deeper in the amniotic mesoderm, the fibroblast layer (4) is composed by a network of rare macrophages and dispersed fibroblast-like mesenchymal cells, responsible for secreting various types of collagens. The final layer, termed the intermediate layer or spongy layer or zona spongiosa (5), lies adjacent to the chorionic membrane and consists primarily of a meshwork of type III collagen (Parry and Strauss, 1998). Its spongy appearance is attributed to the abundant content of proteoglycans and glycoproteins (Malak et al., 1993).

Adjacent but not fused to the amniotic stromal layer, lies the chorion, originating from the blastocyst trophoblast (Moore and Persaud, 1998). The chorionic membrane is composed of a reticular layer, the basement membrane, and the chorionic trophoblastic area (Figure 2, Panel C). The reticular layer of loosely arranged collagen fibers (collagen types I, III, IV, V, and VI, and proteoglycans), separates the amniotic and chorionic mesoderm and contains chorionic mesenchymal stromal cells. The basement membrane, mainly composed by collagen type IV, fibronectin, and laminin, separates the chorionic mesoderm from the chorionic trophoblastic area, rich in proliferating EVT cells, interposed in varying amounts of Langhans’ fibrinoid (Gupta et al., 2015).

In the first trimester, the amnion is incompletely formed, making hard to identify the amniotic layer covering the chorionic plate, and it is not completely attached to the chorion (Menon et al., 2020). By the conclusion of the first trimester, as the amniotic cavity expands, the two membranes initiate adherence to each other (Silini et al., 2020).

### 3.2 Functions of fetal membranes

Throughout pregnancy, the fetal membranes carry out different functions, essential to successful pregnancy outcome, in order to protect and support the growth of the fetus, but also to contribute to the onset of the labor (Menon et al., 2021).

Acting as a fetal-maternal interface, the amnion and the chorion act as a semi-permeable barrier able to compartmentalize potential maternal exposures to various risk factors, respond to local insults (i.e., infection), transport water and nutrients to the fetus, and actively regulate AF flow (Truong et al., 2023), maintaining a homeostatic environment. It has been reported that the amnion is the main membrane in the regulation of AF volume (Beall et al., 2007), by a unidirectional vesicular transcytotic transport of amniotic water with dissolved solutes outward passing through amniotic cells (Ducza et al., 2017). Interestingly, differences in functions of amniotic membrane have been observed depending on the anatomical localization (Weidinger et al., 2020). The fetal membranes participate in the AF composition contributing to a diffusional resistance to water (Hardy et al., 1989). On the other hand, the amniotic membrane lacks blood vessels and nerves and receives nutrition directly through diffusion of the AF (Gupta et al., 2015) and soluble and insoluble factors of AF strongly influence amniotic cells (mainly amniotic epithelial cells) immunophenotype and function during amnion development (Kopaczka et al., 2016).

Serving as a physical barrier, they bear the mechanical stress of fetal movements and AF volume, utilizing a cellular and collagen remodeling mechanism to preserve their structure and function. Moreover, they physically prevent pathogens from entering the amniotic cavity (Truong et al., 2023).

Besides mechanical action, there are also immune modulating functions at the fetal-maternal interface. These result from the production of anti-inflammatory hormones and cytokines, that sustain immunological tolerance and buffer maternal immune cell invasion. Moreover, endocrine capacity of the fetal membranes is related not only to the maintenance of immune homeostasis during pregnancy, but is also related to the induction of several signaling pathways involved in the induction of a pro-inflammatory environment, necessary for labor onset at the end of gestation. Finally, they may also secrete cytokines, chemokines, and antimicrobial peptides as a defense mechanism (Truong et al., 2023).

### 3.3 Membranes features and dimensions

During the first 3 months of pregnancy several placenta components undergo dramatic structural and functional changes in order to fulfill the needs of the developing fetus. The fetal membranes continuously remodel throughout gestation to accommodate the increasing volume of the intrauterine cavity, and this occurs both at the cellular and matrix levels. The mechanical features of the human fetal membranes remain incompletely understood, with limited practical information available for the first trimester. However, based on accessible data across all three trimesters of pregnancy, the amnion emerges as the predominant component of the fetal membranes (Verbruggen et al., 2017), governing the mechanical behavior of the membranes and acting as a structural barrier (Manabe et al., 1991). Conversely, the chorion functions as an immunological buffer, preventing the deterioration of the amnion and shielding the fetus from the maternal immune system (Oyen et al., 2006).

The thickness of the amnion at term varies between 40 and 50 μm (Oxlund et al., 1990) and 111 μm (Oyen et al., 2004; Marom et al., 2016) indicating potential influence from measurement methods. Within amniotic membrane, the thickness of the basement membrane at term is around 2.2 µm (range 1.1–9.3 µm) (Castro et al., 2004), making it one of the thickest basement membranes in all human tissues (Niknejad et al., 2008). It is noteworthy that the chorion layer exceeds the amnion in thickness, measuring at term 413 µm compared to the amnion’s 111 µm (Oyen et al., 2004; Marom et al., 2016) or approximately 180–200 µm compared to the amnion’s 40–50 µm (Oxlund et al., 1990).

Even though the amnion constitutes only 20%–25% of the overall thickness of the fetal membranes, it predominantly influences their mechanical response due to its notable stiffness and strength. The heightened stiffness and strength of the amnion can be attributed to the distribution of collagen and the fibrillar organization within the connective tissue, particularly in the basement membrane and in the compact layer (Malak et al., 1993; Calvin and Oyen, 2007; Jabareen et al., 2009; Gupta et al., 2015). The values for membrane stiffness exhibit significant variability, with measured failure stress ranging between 0.3 and 1.85 MPa at full term. However, these measurements are subject to certain assumptions (Chua and Oyen, 2009). An earlier study (Oxlund et al., 1990) reported apparent elastic modulus of 10.9 ± 0.8 MPa for fresh chorion (180–200 µm) and 47.3 ± 3.0 MPa for fresh amnion (40–50 µm). It is interesting to note that fresh chorion-amnion membrane, which was not separated, showed a stiffness of 15.0 ± 0.9 MPa, in the same conditions of that study. A recent study (Zhang et al., 2021) reported a substantially higher value of 108.57 ± 17.32 MPa, but this is related to the amnion thickness at term corresponding to 425 μm, which is much higher compared to other studies. Therefore, it can be concluded that there remains a scarcity of dependable data regarding the mechanical properties of the membrane, particularly within its physiological range (stresses and strains before rupture). In decellularized full-thickness fetal (amnion and chorion) membrane, the biomechanics assessed by subjecting samples to ball burst, reported an overall mean of 8.43 N (±2.15) force required to break through the full-thickness fetal membrane samples (Wetzell et al., 2024).

Amnion and chorion are permeable to water and semipermeable to other substances (Seeds et al., 1973). Despite the apparent simplicity of measurement, the permeability of the fetal membranes to water *in vitro* has been sporadically studied. In one study, at term human amnion overlying the chorionic plate was examined in a Ussing chamber at 38°C, revealing a measured membrane diffusional permeability to water of 2.2 × 10^−4^ cm/s (Leontic et al., 1979). Another experiment conducted on at term human amniotic membranes, identified an osmotic permeability of 1.5 × 10^−2^ cm/sec. These values are comparable to those observed in renal tubular epithelium (Kuwahara and Verkman, 1988), suggesting that the amnion functions as a “leaky” epithelium with the potential for significant water flux. When both human amnion and chorion were tested, the amnion appeared to be a more effective barrier to the diffusion of water (Hardy et al., 1989). Moreover, chorioamniotic membranes from preterm and term labour have both been shown to be permeable to calcium (Ca) and magnesium (Mg), but the diffusion was lower for both cations in preterm membranes. Specifically, the transport coefficient K for Ca^2+^ was 0.203 h^-1^ in term and 0.0223 h^-1^ in preterm labour; values for Mg^2+^ were −0.017 h^-1^ in term and 0.051 h^-1^ in preterm labour (Lemancewicz et al., 2000).

The medical need to understand and to eventually prevent premature rupture of fetal membranes has driven efforts to characterize their mechanical integrity (Mauri et al., 2015), showing that the force required to puncture the choriodecidua at 37 weeks was lower than that needed for puncturing the amnion (Oyen et al., 2004). Nevertheless, the amnion exhibited greater chemical and mechanical sensitivity, showing variations in response at different physical locations within the same individual (Oyen et al., 2004; Oyen et al., 2005; Oyen et al., 2006). Moreover, recent studies have indicated that repeated mechanical loading, such as that occurring as a result of fetal movement and labor, can impact the microstructure of the fetal membranes (Mauri et al., 2013), leading to a reduction in their toughness. Notably, the superior strength and toughness of the amnion in comparison to the chorion were demonstrated through uniaxial tension to failure tests (Mauri et al., 2013). However, the authors concluded that conventional fracture mechanics-based calculations could not accurately predict the tear resistance of the amnion for defect sizes up to a few millimeters.

## 4 Umbilical cord

### 4.1 Development and structure of the umbilical cord

The human umbilical cord is a long flexible stalk, containing one vein (the umbilical vein) and two arteries (the umbilical arteries) buried within the Wharton’s jelly and enclosed inside a tubular sheath of amnion (Figure 2, Panel B). It forms by week 5 after gestation from the inner cell mass of the blastocyst via a short band of extraembryonic mesoderm known as the body stalk, along with the vitelline duct and allantois (Heil and Bordoni, 2024). Once fully developed, it extends from the umbilicus of the fetus to the center of the placenta, with a length ranging from 50 cm to 60 cm, and a diameter of about 1.5 cm (Weissman et al., 1994; Raio et al., 1999; El Behery et al., 2011). It is not directly linked to the mother’s circulatory system, but it is connected to the placenta. This arrangement ensures the necessary exchange of oxygen, nutrients, and waste products between the maternal and fetal circulations while maintaining their separation (Di Naro et al., 2001; Spurway et al., 2012).

### 4.2 Function, features and dimensions

The human umbilical cord is a conduit between the developing embryo or fetus and the placenta. Its structure allows for the transfer of oxygen and nutrients from the maternal circulation into fetal circulation while simultaneously removing waste products from fetal circulation to be eliminated maternally (Basta and Lipsett, 2024). In particular, the umbilical vein, with a diameter of about 2 mm at weeks 14–15 (Weissman et al., 1994), carries oxygenated, nutrient-rich blood from the placenta to the fetus, with a reported flow of about 443 ± 92 mL/min in the normal (healthy) umbilical cord between 24 and 29 weeks of gestation (El Behery et al., 2011). Instead, the umbilical arteries show a diameter of 1.2 mm on average at weeks 14–15 and carry deoxygenated, nutrient-depleted blood from the fetus to the placenta (Weissman et al., 1994). Whereas umbilical cord intersection into the placenta takes place, these vessels generate chorionic arteries that extend towards the villi (Wang and Zhao, 2010).

## 5 Maternal and fetal fluid dynamic exchanges

### 5.1 Feto-placenta vasculature

The structure and the functions of the feto-placental vasculature are dynamic during gestation and metabolic communication throughout pregnancy is achieved by bringing fetal and maternal blood into close proximity, avoiding direct contact (Clark et al., 2015). The understanding of placental fluid dynamics and associated transport mechanisms is crucial for elucidating the connection between the complex structure and functions of the placenta (Rainey and Mayhew, 2010; Jensen and Chernyavsky, 2019; Bowman et al., 2021). It provides also valuable insights into conditions like preeclampsia and diabetes, where the placenta architecture and function are compromised (Rainey and Mayhew, 2010; Majali-Martinez et al., 2021).

**FIGURE 3:**
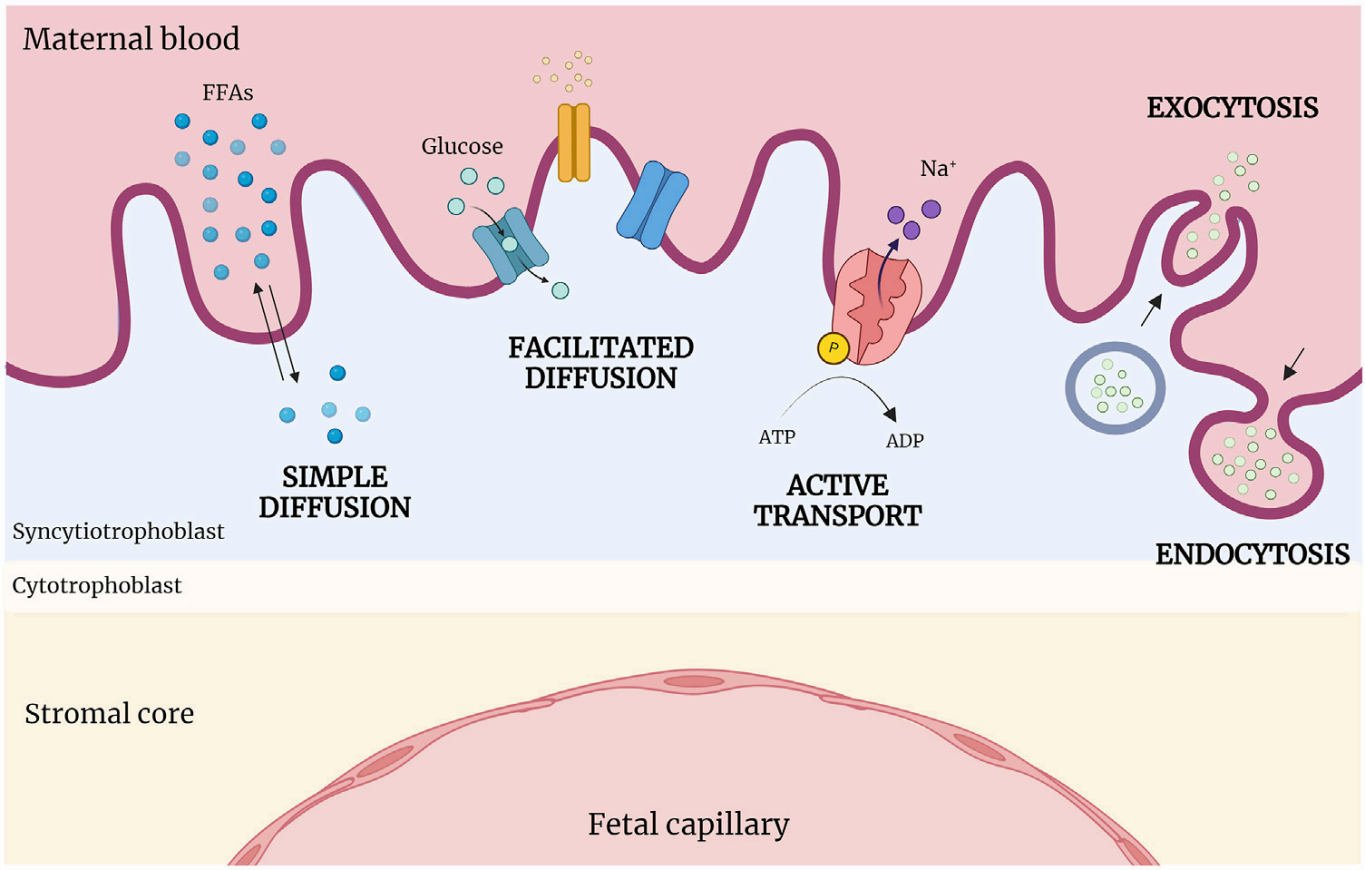
Abridged scheme of transports across placenta. At the fetal-maternal interface, different transport mechanisms operate based on the biochemical properties of molecules. Simple diffusion occurs following the concentration gradient; in contrast, facilitated diffusion requires the presence of specific channels and pores. Active transport moves substances against their concentration gradient using energy (ATP), while macromolecules enter cells via endocytosis or exocytosis (vesicular transport). (Created with BioRender.com).

Different types of transport mechanisms occur at fetal-maternal interface level, based on biochemical characteristics of molecules and substances (Figure 3). The diffusion process across membranes follows, broadly, two pathways, a lipophilic route (for lipid-soluble molecules), and a hydrophilic route (for lipid-insoluble molecules), depending on the concentration gradient (simple diffusion). Specifically, particles diffuse from the side with the higher concentration to the side with the lower concentration, until reaching a balanced condition without energy dispersion (i.e. oxygen (O_2_), carbon dioxide (CO_2_), fats). Instead, glucose, water and other substances transit with the help of specific channels and pores according to the concentration gradient (facilitated diffusion). Cells can transport substances also against their concentration gradient (sodium/potassium (Na/K) or calcium (Ca); adenosine triphosphate (ATP)) through energy consumption (active transport), or allow the entrance of macro-molecules (immunoglobulin) by endocytosis or exocytosis (vesicular transport) (Desforges and Sibley, 2010; Lager and Powell, 2012).

#### 5.1.1 Vasculature structure and interface features

The placenta is characterized by two separate circulatory systems for blood supplies: (i) the maternal-placental (uteroplacental) blood circulation, and (ii) the feto-placental blood circulation (Wang and Zhao, 2010).

(i) The uteroplacental circulation begins around week 7, with a limited maternal blood flow into IVS, that significantly increased its flux rate around 13 weeks (Roberts et al., 2017). As previously described, the genesis of maternal-placental blood flow is not completely understood, but it is a dynamic and progressive process, characterized by the invasion of the spiral arteries (SAs) by trophoblasts (Burrows et al., 1996; Jaffe et al., 1997; Meekins et al., 1997; James et al., 2018a; Allerkamp et al., 2021). Vasculogenesis during the first trimester is associated with the increased number of vessels in the peripheral area of the chorion, that also entails a decrease in the stromal counterpart. From week 5, the healthy placenta presents well-luminized vessels, whose presence on the villous stromal area increases from 0.5% at week 5 to 2.5% at week 10 of gestational age (te Velde et al., 1997). The use of 3D power Doppler (PD) ultrasonography and a skeletonization algorithm during vascularization development revealed a physiological remodeling of the arcuate, radial, and spiral arteries, that progressively increased their dimensions 2.6- to 4.3-fold between 6 and 20 weeks of gestation (Allerkamp et al., 2021).

Once the uteroplacental circulation is completely established, maternal blood reaches the placenta through the endometrial arteries (about 120 SAs at term) (Mercé et al., 2009; Cunningham et al., 2018), and perfuses IVS, and flows around the villi, where the exchange barrier consists of the epithelial cell layers (SCTB and CTB), basement membrane and connective tissue, and the fetal capillary endothelium (Benirschke et al., 2012). At this level, the exchange of O_2_ and nutrients occurs with fetal blood, while the deoxygenated blood returns to the maternal circulation through uterine veins.

It is reported that placental blood flow is increased throughout pregnancy, with an average perfusion rate of about 129 ± 61 mL/min/100 mL (dynamic contrast enhanced magnetic resonance imaging) (Deloison et al., 2021) –176 ± 24 mL/100 g/min (echo planar imaging) (Gowland et al., 1998) and a volume of about 600–700 mL/min (80% of the uterine perfusion) at term (Wang and Zhao, 2010).

Due to the low-resistance nature of uteroplacental vessels, the maternal arterial pressure is responsible for maintaining the maternal-placental blood flow (Cunningham et al., 2018). However, blood pressure is not constant, instead a gradient is necessary to ensure the efficient and effective perfusion of the intervillous space. Indeed, the pressure undergoes a significant gradient from uterine arteries to intervillous spaces. It starts at around 80–100 mmHg in uterine arteries, decreases to approximately 70 mmHg in spiral arteries, and further drops to 10 mmHg within the intervillous space. This pressure gradient facilitates a thorough exchange of the intervillous space blood, occurring two to three times per minute, supporting the vital functions of the placenta for optimal fetal development (Wang and Zhao, 2010).

(ii) The feto-placental circulation occurs through the presence of the umbilical cord, which serves as the connecting link between the placenta and the fetus. As mentioned above, the umbilical vein carries oxygenated, nutrient-rich blood from the placenta to the fetus. Instead, the umbilical arteries carry deoxygenated, nutrient-depleted blood from the fetus to the placenta, and they branch out into the chorionic plate, reaching the villi. Within the villi, these arteries supply the cotyledons, spreading out in a pattern of disperse-type branching. In each cotyledon, approximately 30–60 branches can be identified, with calibers of 0.1–0.6 mm and lengths of 15–25 mm (Wang and Zhao, 2010). The blood pressure is typically around 20 mmHg in the umbilical vein and averages about 50 mmHg in the umbilical arteries. Nevertheless, arterial blood pressure falls to 30 mmHg in the villi capillaries, being however always greater than that within the intervillous space, to avoid the fetal vessels collapse (Wang and Zhao, 2010).

#### 5.1.2 Functional aspects of the feto-placenta vasculatures

The fetal-maternal interactions begin to take shape in the first weeks of development and undergo continuous changes and adaptations during the course of pregnancy (Reynolds et al., 2010; Clark et al., 2015; De Vos et al., 2024). From the week 4 of gestation, the fetal circulation patterns are formed and upon optimal establishment of maternal blood flow into intervillous space, haemotrophic support of the fetus can occur across the villous tree barrier. However, due to the rapid growth and development of the fetus, a substantial morphological remodeling of villi takes place during the second trimester to optimize the supply of nutrients and oxygen. Once established, the maternal and fetal circulations will never directly come into contact, and the exchange of substances occurs mainly via passive diffusion across the SCTB layer. Maternal and fetal flows travel in the same direction, establishing the so-called concurrent exchange, and metabolites and water maternal-fetal transfer rate exceeds the fetal-maternal transfer rate over the course of pregnancy, thereby controlling fetal growth by influencing either unidirectional flux (McNanley, 2008).

#### 5.1.3 Vascular metabolite exchanges

As anticipated, several and very different transport processes occur at placental level, due to the multiplicity of molecules and substances exchanged (Figure 3).

Oxygen readily crosses the placenta by passive diffusion which transfer mainly depends on the O_2_ partial pressure (pO_2_) gradient between maternal blood in the intervillous space and fetal blood. In fact, at the fetal-maternal interface, the CO_2_ taken up by the maternal blood lowers the pH. This favors the release of O_2_ to the fetus, which at the same time releases CO_2_ in the blood, raising the pH, which in turn favors the fetal uptake of oxygen. Oxygen can diffuse across the SCTB only in its diffused form -not bound to hemoglobin-, and differences in maternal and fetal hemoglobin (HbA and HbF, respectively) affect the delivery and uptake of fetal O_2_ (Acharya and Sitras, 2009; Mushambi, 2009). The amount of hemoglobin binding sites occupied by O_2_ is identified as the percentage of saturation, which, along with pO2, provide information about the O_2_ concentration in the blood.

Under normal pregnancy conditions, hemoglobin saturation in the maternal artery is near 100%, while pO_2_ in the maternal blood bathing the placental villi is < 20 mmHg until 10–12 weeks of gestation, rising to 40–80 mmHg and remaining in this range throughout the second and third trimesters (Schaaps et al., 2005; Tuuli et al., 2011). Thus, the human placenta develops initially in a low O_2_ environment, that increases when the intervillous circulation is established (10–12 weeks). Higher O_2_ content in the first weeks of pregnancy could affect fetal development, especially for the elevated production of reactive O_2_ species in the absence of proper antioxidant mechanisms, not yet established (Jauniaux et al., 2003; Al-Gubory et al., 2010; Matsubara et al., 2010; Hussain et al., 2021). From the second and third trimester of pregnancy, saturation of O_2_ in umbilical artery measures approximately 62.1%–70.4%, with a pO_2_ from 24 to 29 mmHg, CO_2_ pressure (pCO_2_) from 35 to 46 mmHg and pH 7.35–7.40 (Nicolaides et al., 1989), that could be compared to the solubility and saturation values shown earlier (Rooth et al., 1961; Kiserud and Acharya, 2004; Acharya et al., 2016). These values decrease with the advancing of gestation (Nicolaides et al., 1989; Lazarević et al., 1991; Acharya et al., 2016). Once oxygenated, nutrient-rich blood returns to the fetus by the umbilical vein, with a continuous and non-pulsatile flow. Saturation of O_2_ in the umbilical vein is approximately 80%–88%, pO_2_ 44–46 mmHg, pCO_2_ 33–34 mmHg and pH 7.425–7.430 at mid-gestation, with a decrease in the O_2_ saturation to 67%–75%, pO_2_ 30–34 mmHg, pCO_2_ 36–37 mmHg and pH 7.38–7.41 at term (Gilbert et al., 1991; Acharya et al., 2016). During gestation, the level of O_2_ saturation in the umbilical vein decreases. This decrease is balanced by an increase in hemoglobin concentration, necessary to maintain the O_2_ content at a constant level. In the developing intervillous space, pO_2_ levels increase during the initial phases of gestation until 40 mmHg (Soothill et al., 1986), with a decline from the second to the third trimester. The calculated mean value at term was 39,9 mmHg (Sjöstedt et al., 1960; Rooth et al., 1961; Nye et al., 2018).

Water and electrolyte diffusion is important to maintain the correct ion composition within cell, which influences cellular turgor pressure, pH and metabolic activity. Water molecules easily diffuse into the placenta along an osmolar gradient, followed by electrolytes. Water is transferred across the hemochorial placenta through both the paracellular and transcellular routes, and its transfer may be facilitated by integral membrane water channel proteins (i.e. aquaporins) (Sha et al., 2011). During the early stages of fetal development, the osmotic process occurs in all three compartments: maternal blood, fetal blood, and amniotic fluid (AF). As pregnancy progresses, the fetal skin becomes more keratinized and less water is transferred from the fetus to the AF through osmosis, with 95% of water exchange between mother and fetus occurring through the placenta (Hutchinson et al., 1959; Dale et al., 1985; Stulc, 1997; Martínez and Damiano, 2017; Damiano, 2020). Although large amounts of water move across the placenta, the net transfer is relatively balanced due to similar solute concentrations on both sides. The amount of water exchanged per hour increases from about 100 mL/h around week 20 to 3,600 mL/h at term (Hutchinson et al., 1959).

Iron and calcium only move from mother to fetus by active carrier-mediated transport, while sodium and potassium flow occurs through Na:K pumps, located on the fetal surface. The intracellular fluid contains always a higher level of potassium and a lower level of sodium, and this relationship should be maintained also at the placenta level. Concentrations of sodium, potassium, calcium, chlorine and phosphate are accurately balanced and are comparable between the mother and the fetus (McNanley, 2008).

Amino acids and peptides are too large to pass through the placental barrier and are transferred from mother to fetus by active transport. There are several transporter proteins specific for anionic, cationic, and neutral amino acids. Many of these proteins’ co-transport amino acids with sodium: the transport of sodium down its concentration gradient drags amino acids into the cells. Hormones can influence this transit across the placenta barrier, with a passage 2–3 times higher in the fetus as in the mother (Pepe and Albrecht, 1995; Sha et al., 2011). Water-soluble vitamins pass easily through the placental membrane by active carrier-mediated transport, while the amount of the fat-soluble vitamins (A, D, E and K) in the fetal circulation is kept quite low (Sawaya, 2010).

Maternal glucose forms its main source of energy since the fetus has very little capacity for gluconeogenesis. Glucose passes across the placenta by passive diffusion, but it is insufficient to meet the needs of the fetus, therefore the transfer of glucose across the placenta is facilitated by specific transporters (Desforges and Sibley, 2010). The demonstrated linear relationship between maternal and fetal glucose concentrations underscores the importance of maternal glucose levels in meeting the energy needs of the developing fetus throughout pregnancy (Bozzetti et al., 1988; Hay, 2006). In fact, it was reported that in growth-retarded cases, the fetal glucose was significantly lower (mean = 2.7 mmol/L) than in normal pregnancies (mean = 3.5 mmol/L) (Nicolini et al., 1989).

Free fatty acids (FFAs) and glycerol are transferred from mother to fetus mainly by simple diffusion and through fatty acid binding proteins. Since fatty acids are important for the synthesis of compounds involved in cell signaling and for the production of fetal phospholipids, biological membranes, and myelin, pregnancy is generally marked with hyperlipidemia involving lipoproteins, triacylglycerols, and FFAs (Darmady and Postle, 1982; Duttaroy and Basak, 2021), providing the fetus with easy access to FFAs (Warth et al., 1975; Hanebutt et al., 2008; Cetin et al., 2009). Accordingly, plasma FFA concentrations rise rapidly during the third trimester, thus representing the main class of lipids crossing the placenta (Magnusson-Olsson et al., 2006). Phospholipids increase in plasma level by approximately 65% during the third trimester as compared with first trimester of pregnancy (Punnonen, 1977; Wijendran et al., 1999), while phospholipid-containing arachidonic acid and docosahexaenoic acid increase on average by ∼40% from the first trimester (10 weeks) to the time of parturition. Finally non-essential unsaturated fatty acids increase more than 65% with the progression of gestation. Ultimately, a strong linear association was observed between the maternal docosahexaenoic acid (DHA) level and the phospholipid contents in umbilical cord plasma (Larqué et al., 2006; Calder, 2016).

### 5.2 Amniotic fluid: Features and function

The amniotic fluid (AF) is a clear and watery fluid, formed during the first trimester of pregnancy from fetal and possibly maternal compartments. After the 4th week of pregnancy, the amniotic cavity is filled with AF, and completely surrounds the embryo and forms a protected, enclosed space where the embryo is free to move. AF constitutes a reservoir of fluid, nutrients and growth factors that are essential for fetal growth, and, together with the fetal membranes, protects the fetus from mechanical trauma, avoiding compression with the uterine wall and possible crushing of the umbilical cord. Moreover, due to its antibacterial properties, it plays an important defense mechanism also toward infectious agents (ten Broek et al., 2013).

The large portion of AF comes from the fetus itself, while the other one is filtered from the maternal plasma via the amniotic epithelium basing on hydrostatic and osmotic forces, and then reached the amniotic cavity (Capurro et al., 1989; Beall et al., 2007). From the second trimester, when the fetal skin becomes more keratinized (Tan et al., 2014), the fetal contribution to the AF formation is given by urination (Abramovich, 1970; Fisher, 2008). Its volume changes during the course of pregnancy, correlating in the first half of pregnancy with fetal weight, with an average value of 60 mL at 12 weeks and 175 mL from 16 weeks (Elliott and Inman, 1961; Abramovich, 1970; Brace and Wolf, 1989).

From published studies, it is possible to compare some parameters of interest regarding the composition of the AF at different stages of pregnancy; however, for some of them, comparisons may be challenging due to the absence of available data (Campbell et al., 1992). The concentration of total proteins in the AF is higher in early gestation compared to the mid-late period. These differences can be attributed to the fact that the fetus initiates swallowing the AF, which serves as a form of clearance for AF proteins. Variations in this parameter have been associated with abnormal fetal development (Tisi et al., 2004). One of the parameters that frequently changes is creatinine, and its elevation beyond benchmark values has been related with maternal renal insufficiency (Williams et al., 1981; Oliveira et al., 2002). Moreover, it has been considered as fetal maturity index, although with contrasting outcomes (Ciani et al., 1980; Williams et al., 1981). Glucose levels increase during gestation (2.8 ± 0.5 nmol/L at the end of the first trimester) (Jauniaux et al., 1994), and are considerably lower when compared to values reported in the blood (Romero et al., 1990; Boris Draznin, 2022).

Electrolyte levels in the AF through gestation are comparable to those present in the blood stream, with some minimal differences. Indeed, sodium (125.6 mmol/L), potassium (4.5 mmol/L) and chlorine (109.3 mmol/L) ions in the AF at term are similar to physiological concentrations, while calcium (2.0 mmol/L) is slightly lower (Demir et al., 1992). Even phosphate (0.71 mmol/L), bicarbonate (38 ± 6 mmol/L) (Jauniaux et al., 1994) and magnesium (1.65 ± 0.16 mg/dL) (Bocos Terraz et al., 2011) are slightly lower compared to blood concentrations in the first phases of pregnancy (8–18 weeks). The overall pH detected in AF is neutral, specifically 7.2 ± 0.1 (Yılmaz Semerci et al., 2020). All these parameters support the concept that AF is a medium which allows the maintenance of a physiological balance during fetus growth, with some alterations due to fetal development.

## 6 Concluding remarks

The study of the exact structure and functionality of the human placenta in the first trimester is challenging due to ethical constraints on using first-trimester specimens and technological limitations. In order to emulate the human placenta biology, a clear view on the biophysical and mechanical characteristics is necessary to design organotypic *in vitro* models including the fluidics that majorly determine placenta functionality. Such models will then allow to model diseases or toxicities that otherwise are not amenable to interventional studies.

This manuscript underscores that current data on the biomechanics of placental tissues, the mechanisms of substance transport, and blood circulation parameters - especially in the early stages of pregnancy - remain insufficient. Furthermore, it points out how the existing data frequently show inconsistencies due to variations in methodologies utilized for analysis.

In the context of this review, we have presented information on the first trimester of healthy placental development and biophysical parameters to the best of our knowledge, along with available data concerning more advanced stages of pregnancy, particularly at term, where more information is available, and which can provide valuable insights that can be extrapolated to enhance our understanding of the first trimester.

Due to the pivotal role that the intricate cellular and molecular interactions at the fetal-maternal interface play in shaping pregnancy outcomes, further research into these interactions is essential. Advancing our knowledge of placental development, immune defence mechanisms, and adaptations critical for maintaining a healthy pregnancy will improve prenatal care, diagnostics, and treatments. Ultimately, this will lead to enhanced maternal and fetal health outcomes.
